# Deficiency of eNOS exacerbates early-stage NAFLD pathogenesis by changing the fat distribution

**DOI:** 10.1186/s12876-015-0409-9

**Published:** 2015-12-17

**Authors:** Yuichi Nozaki, Koji Fujita, Koichiro Wada, Masato Yoneda, Yoshiyasu Shinohara, Kento Imajo, Yuji Ogawa, Takaomi Kessoku, Makoto Nakamuta, Satoru Saito, Naohiko Masaki, Yoji Nagashima, Yasuo Terauchi, Atsushi Nakajima

**Affiliations:** 1 Department of Gastroenterology, Yokohama City University Graduate School of Medicine, 3-9 Fuku-ura, Kanazawa-ku, 236-0004 Yokohama, Kanagawa, Japan; 2 Department of Gastroenterology, National Center for Global Health and Medicine, 1-21-1, Toyama, Shinjuku-ku, 162-8655 Tokyo, Japan; 3 Department of Pharmacology, Graduate School of Dentistry, Osaka University, 1-8 Yamadaoka, 565-0871 Suita, Osaka, Japan; 4 Department of Gastroenterology, Kyushu Medical Center, National Hospital Organization, 1-8-1, Jigyohama, Chuo-ku, 810-8563 Fukuoka, Japan; 5 The Research Center for Hepatitis and Immunology, National Center for Global Health and Medicine, 1-7-1, Konodai, 272-8516 Ichikawa, China, Japan; 6 Department of Molecular Pathology, Yokohama City University Graduate School of Medicine, 3-9 Fuku-ura, Kanazawa-ku, 236-0004 Yokohama, Kanagawa, Japan; 7 Department of Surgical Pathology, Tokyo Women`s Medical University, 8-1, Kawada-cho, Shinjuku-ku, Tokyo, 162-8666 Japan; 8 Department of Endocrinology and Metabolism, Yokohama City University Graduate School of Medicine, 3-9 Fuku-ura, Kanazawa-ku, 236-0004 Yokohama, Kanagawa, Japan

**Keywords:** NO, NAFLD, Hepatic tissue blood flow, Fat distribution, Obese mice

## Abstract

**Background:**

Although many factors and molecules that are closely associated with non-alcoholic fatty liver disease (NAFLD)/non-alcoholic steatohepatitis (NASH) have been reported, the role of endothelial nitric oxide synthase (eNOS)-derived nitric oxide (NO) in the pathogenesis of NAFLD/NASH remains unclear. We therefore investigated the role of eNOS-derived NO in NAFLD pathogenesis using systemic *eNOS*-knockout mice fed a high-fat diet.

**Methods:**

*eNOS*-knockout and wild-type mice were fed a basal diet or a high-fat diet for 12 weeks. Lipid accumulation and inflammation were evaluated in the liver, and various factors that are closely associated with NAFLD/NASH and hepatic tissue blood flow were analyzed.

**Results:**

Lipid accumulation and inflammation were more extensive in the liver and lipid accumulation was less extensive in the visceral fat tissue in *eNOS*-knockout mice, compared with wild-type mice, after 12 weeks of being fed a high-fat diet. While systemic insulin resistance was comparable between the *eNOS*-knockout and wild-type mice fed a high-fat diet, hepatic tissue blood flow was significantly suppressed in the *eNOS*-knockout mice, compared with the wild-type mice, in mice fed a high-fat diet. The microsomal triglyceride transfer protein activity was down-regulated in *eNOS*-knockout mice, compared with wild-type mice, in mice fed a high-fat diet.

**Conclusions:**

A deficiency of eNOS-derived NO may exacerbate the early-stage of NASH pathogenesis by changing the fat distribution in a mouse model via the regulation of hepatic tissue blood flow.

## Background

Non-alcoholic fatty liver disease (NAFLD) is one of the most important causes of chronic liver disease and is associated with systemic insulin resistance (IR) and metabolic syndrome. NAFLD includes non-alcoholic fatty liver (NAFL) and non-alcoholic steatohepatitis (NASH); NAFL represents the first phase of NASH, which is characterized by steatosis, and can then develop into fatty liver disease with associated inflammation [1]. NASH is thought to lead to liver fibrosis, cirrhosis, and hepatocellular carcinoma, resulting in increased morbidity and mortality. The pathogenesis of NAFLD remains to be clarified fully.

We previously reported that several potential mechanisms are involved in the pathogenesis of NAFLD [2–4]. However, other factors associated with NAFLD progression remain to be determined.

Nitric oxide (NO) is a gas produced by nitric oxide synthase (NOS) enzyme [5], and three major isoforms of NOS are known to exist: neuronal NOS (nNOS), inducible NOS (iNOS), and endothelial NOS (eNOS). eNOS-derived NO is known to have important endothelial functions, including the regulation of vascular tone and regional blood flow [6]. On the other hand, liver steatosis has been shown to be associated with sinusoidal narrowing and a reduction in sinusoidal flow; furthermore, microvascular changes may contribute to progressive liver injury in metabolic forms of steatohepatitis [7]. Chronic NOS inhibition has been reported to accelerate NAFLD progression [8]; however, the roles of eNOS and generated NO in the pathogenesis of NAFLD/NASH have not yet been fully elucidated in *eNOS* knockout (*eNOS-/-*) mice fed a high-fat diet (HFD).

In this study, we investigated the role of eNOS-derived NO in the pathogenesis of NAFLD using systemic *eNOS*-knockout mice fed an HFD.

## Methods

### Animal treatment and procedures

We purchased a C57BL/6J backcrossed C57BL/6J-Nos3tm1Lau colony from Jackson Labs (Bar Harbor, ME 04609, USA) to create male congenic wild-type (*eNOS+/+*) and *eNOS* knockout (*eNOS-/-*) mice. Mice were allowed free access to food and tap water throughout the acclimatization and experimental periods.

Beginning at 6 weeks of age, each strain of mice was fed a basal diet (BD) or an HFD. Thirty-two mice were divided into 4 groups: (1) *eNOS+/+* and BD; (2) *eNOS-/-* and BD; (3) *eNOS+/+* and HFD; and (4) *eNOS-/-* and HFD. After a 12-week experimental period, eight mice in each group were examined after being subjected to an overnight fast. The HFD consisted of high-fat diet 32, containing 20 % protein, 60 % fat, and 20 % carbohydrate, in powdered form from Japan CLEA (Tokyo, Japan), while the BD consisted of MF, containing 22 % protein, 6 % fat, and 47 % carbohydrate, from Oriental Yeast Co., Ltd. (Tokyo, Japan). The precise contents of these feeds have been described previously [4]. All the animals were treated humanely according to the guidelines of the National Institutes of Health and the AERI-BBRI Animal Care and Use Committee. The animal protocols were approved by the Yokohama City University Medical School guidelines for the care and use of laboratory animals. This study was conducted by the approval of the institutional review board at the Yokohama City University Medical School.

### Measurement of plasma and serum biochemical markers

Serum alanine aminotransferase (ALT) was measured using Spotchem SP-4410 (Arklay Co., Kyoto, Japan). The total serum cholesterol (Chol) and triglyceride (TG) levels were analyzed using an online dual-enzymatic method for the simultaneous quantification of cholesterol and TG using high-performance liquid chromatography according to a previously described procedure [4, 9, 10]. The fasting plasma glucose level and the serum levels of fasting insulin, leptin, adiponectin, and non-esterified fatty acid (NEFA) were determined using a Glutest Pro kit (Sanwa Kagaku Kenkyusyo Co., Nagoya, Japan), an Ultra Sensitive Insulin ELISA kit (Biochemical Research Laboratory, Morinaga Milk Industry Co., Tokyo, Japan), an ELISA Mouse Leptin kit (Biochemical Research Laboratory, Morinaga Milk Industry Co., Tokyo, Japan), a Mouse/Rat Adiponectin ELISA kit (Otsuka Pharmaceutical Co., Tokyo, Japan), and a NEFA C-Test kit (Wako Pure Chemical Industries Co., Osaka, Japan), respectively. The blood IR was estimated using the homeostasis model assessment of IR (HOMA-IR) and the following equation: IR = fasting plasma glucose level (mg/dL) × fasting serum insulin level (ng/mL)/22.5.

### Insulin tolerance test (ITT)

Mice were fed *ad libitum* prior to being subjected to a fast immediately before the start of the study. The mice were intraperitoneally challenged with 0.75 mU/g body weight of human insulin (Novolin R; Novo Nordisk, Denmark). Blood samples were then collected to measure the glucose levels at 0, 30, 60, 80, 100, and 120 min after the insulin injection [11].

### Measurement of liver triglyceride content

The liver samples were homogenized in 50 mM Tris/HCl buffer, pH7.4, containing 150 mM NaCl, 1 mM EDTA, and 1 mM PMSF. The liver TG levels were analyzed enzymatically using a diagnostic kit (Infinity™; Thermo DMA, Arlington, TX, USA).

### Liver MTP activity assay

MTP activity was measured using an MTP assay kit (Roar Biochemical, New York, NY, USA), according to a previously described method [12, 13].

### CT scanning

In the 12-week model, the abdomen of each mouse was scanned prior to sacrifice using an X-ray computed tomography (CT) system (eXplore Locus; GE Healthcare Bio-Sciences GK, Tokyo, Japan) developed for small experimental animals [14]. The mice were anesthetized for the duration of the scan, and non-contrast CT scanning was performed.

### Measurement of liver/spleen ratio of CT values and visceral fat volume

The hepatic attenuation index was obtained using non-contrast CT images to determine the liver-to-spleen attenuation ratio [15]. The inverse of the liver-to-spleen density ratio was regarded as an indicator of the degree of fat infiltration in the liver [16]. The visceral fat volume was quantified using CT imaging [17, 18].

### Determination of Hepatic Tissue Blood Flow

At 12 weeks, each mouse was anesthetized and a laparotomy was conducted to determine the hepatic tissue blood flow at several points located 1 mm from the edge of the right or left lobe using a noncontact-type laser Doppler blood flow meter (OMEGAFLO FLO-N1; OMEGAWAVE Inc., Tokyo, Japan) [19, 20]. The measurements were performed at 3 different points in each of the right and left lobes. The mean of the three values for each lobe was calculated, and the resulting values were expressed as a percentage of the value obtained in the *eNOS+/+* and BD group.

### Liver histopathological and immunohistochemical evaluations

Liver samples were excised and embedded in Tissue-Tek OCT compound (Sakura Finetek USA Inc., Torrance, CA, USA) and paraffin for the histological analysis. Formalin-fixed and paraffin-embedded sections were processed routinely using hematoxylin and eosin (H&E), myeloperoxidase (MPO), naphthol AS-D chloroacetate esterase [21] and Sirius-red. To evaluate the fat deposition, the OCT-embedded samples were stained with oil-red O.

### Liver histology and scoring systems

All the histopathological findings were scored by the same pathologists (Y.N. and S.M.), who were unaware of the treatments that the animals had received. The histological features were grouped into 3 broad categories (steatosis, inflammation, and fibrosis) to enable the use of the NAFLD activity score (NAS) [22]. The evaluation protocol, which has been described previously [23], is detailed in Table 3.

### RNA isolation and reverse transplantation

Total RNA was isolated from the samples using an RNeasy Mini kit (Qiagen GmbH, Hilden, Germany; Cat No. 74126); reverse transcription was then performed to produce cDNA using a TaqMan Gold RT-PCR Kit (Applied Biosystems, Foster City, CA, USA) according to the manufacturer’s instructions, as previously described [4].

### Quantification of gene expressions using real-time RT-PCR

The hepatic mRNA levels of several markers, as well as the housekeeping gene β-actin, in the liver tissues were determined using fluorescence-based real-time RT-PCR and an ABI PRISM 7700 Sequence Detection System (Applied Biosystems, Foster City, CA, USA). Real-time RT-PCR was performed using the TaqMan and Power SYBR^Ⓡ^ Green PCR Master Mix reagent, according to the manufacturer’s instructions (Applied Biosystems, Foster City, CA, USA). The values were normalized to the expression level of the endogenous control, β-actin. The gene expression ratio was determined using data from the *eNOS+/+* and BD group mice as the control group. The probe and primer pair specific for β-actin were purchased from Applied Biosystems. The primer sequences of sterol regulatory element binding protein-1c (SREBP-1c), peroxisome proliferators-activated receptor alpha1 (PPAR-α1), nNOS, and iNOS are listed in Table 1.

**Table 1:** Primer sequences used for real-time PCR analysis

Gene	Primer sense	Primer antisense
SREBP-1c	CAGCTATTGGCCTTCCTCAG	CCTGGACCATTTTAGCCTCA
PPAR-α	GTCCTCAGTGCTTCCAGAGG	GGTCACCTACGAGTGGCATT
nNOS	TCCTAAATCCAGCCGATCGA	TCATGGTTGCCAGGGAAGAC
iNOS	AGAGAGATCCGATTTAGAGTCTTGGT	TGACCCGTGAAGCCATGAC

### Statistical analysis

The statistical analyses were performed using SPSS for Windows, version 12 (IBM Co. Armonk, NY, USA). All the results were expressed as the mean ± SEM. Statistical comparisons were made using the Student *t*-test or Scheffe’s method after an analysis of variance (ANOVA). A value of *P* < 0.05 was considered statistically significant.

## Results

### HFD feeding induced the phenotype of metabolic syndrome and the pathogenesis of early-stage NASH

**Fig. 1:**
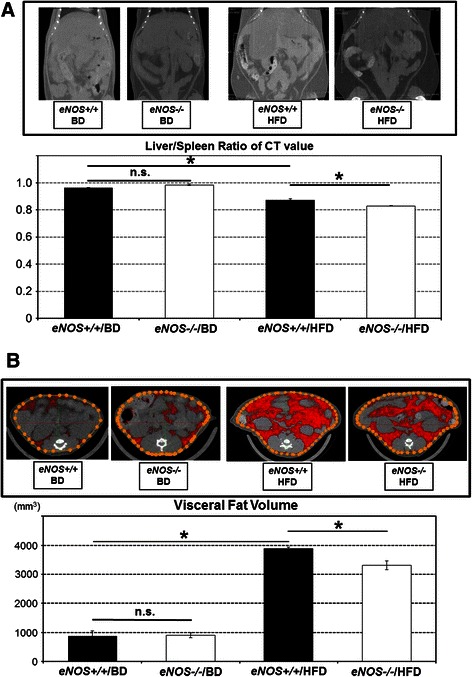
Analysis of liver weight, lipid deposits in the liver, and visceral fat volume. **a** The liver-to-spleen attenuation ratio obtained using non-contrast CT images showed that hepatic steatosis was significantly severer in the *eNOS-/-*/HFD mice than in the *eNOS+/+*/HFD mice at 12 weeks. **b** The visceral fat volume quantified in the CT imaging of the *eNOS-/-*/HFD mice was significantly lower than that of the *eNOS+/+*/HFD mice at 12 weeks. (Data were expressed as the mean ± SEM. *P < 0.05, represents a significant difference; n.s. represents no significant difference.)

**Fig. 2:**
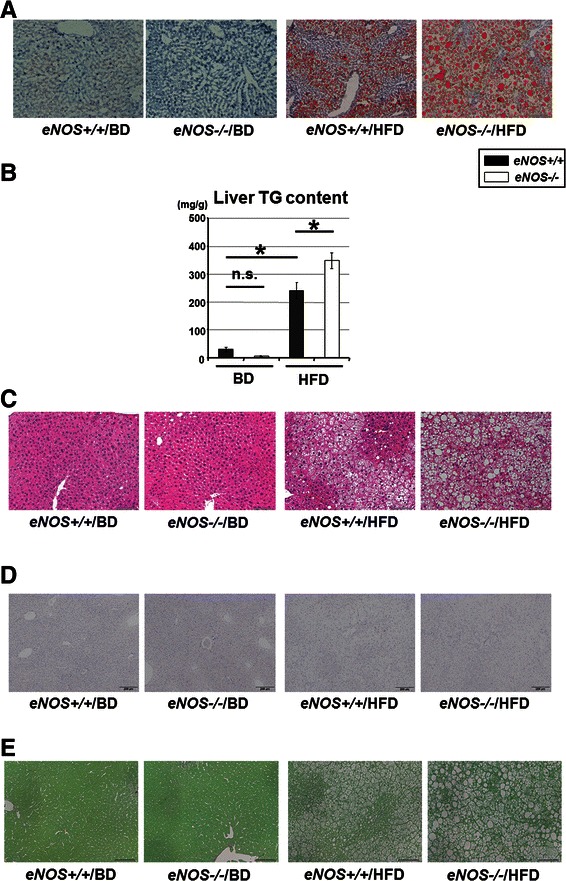
Analysis of liver steatosis and inflammation. **a** Oil-red O staining (red color) shows lipid deposits in liver samples. At 12 weeks, macrovesicular steatosis was visible in the *eNOS-/-*/HFD mice and microvesicular steatosis was visible in the *eNOS+/+*/HFD mice. Scale bar, 200 μm. **b** The liver TG content was significantly higher in the *eNOS-/-*/HFD mice than in the *eNOS+/+*/HFD mice. The liver TG content was significantly higher in the *eNOS+/+*/HFD mice than in the *eNOS+/+*/BD mice, which were regarded as the control group. **c** Liver samples stained using H&E. An evaluation of the number of inflammatory foci per field and the amount of ballooning revealed that the *eNOS-/-*/HFD mice exhibited severer liver inflammation than the *eNOS+/+*/HFD mice at 12 weeks. Scale bar, 200 μm. **d** and **e** Liver samples stained using myeloperoxidase (**d**) and Sirius red (**e**) showed no significant positive findings. (Data were expressed as the mean ± SEM. *P < 0.05, represents a significant difference; n.s. represents no significant difference.)

After 12 weeks of HFD feeding, the *eNOS+/+* and HFD mice had significantly higher body weights, liver weights, and visceral fat weights than the control mice (*eNOS+/+* and BD) (Table 2). The *eNOS+/+* and HFD mice showed signs of insulin resistance and dyslipidemia (Table 2 and Fig. 4a), thus demonstrating the phenotype of metabolic syndrome. Furthermore, hepatic steatosis and very mild inflammation were observed in the *eNOS+/+* and HFD mice, which were considered to have early-stage NASH (Table 3 and Figs. 1a, 2a-d). The reason for the negative findings of the liver tissue MPO staining examination might be related to the model used in this study for early-stage NASH, which showed very mild inflammation, such as very small inflammatory foci detected in the H-E stained samples (Fig. 2d). No apparent signs of liver fibrosis were observed in any of the groups (Fig. 2e).

**Table 2:** Characteristics of mice in the 12-week model

	12-week model			
	*eNOS+/+*BD	*eNOS-/-*BD	*eNOS+/+*HFD	*eNOS-/-*HFD
Number of animals	8	8	8	8
Body weight (g)	28.1 ± 1.9	27.2 ± 2.4	42.8 ± 3.1*	41.7 ± 2.4
Liver weight (g)	1.2 ± 0.1	1.2 ± 0.2	2.3 ± 0.6*	2.8 ± 0.5§
Visceral fat weight (g)	0.6 ± 0.1	0.7 ± 0.3	2.0 ± 0.3*	1.2 ± 0.3§
Liver weight/body weight ratio (%)	4.2 ± 0.3	4.3 ± 0.6	5.2 ± 1.0*	6.7 ± 1.0§
Visceral fat weight/body weight ratio (%)	2.1 ± 0.4	2.4 ± 1.0	4.7 ± 1.0*	3.0 ± 0.6§
Serum ALT (IU/L)	9.2 ± 0.8	17.5 ± 9.3	74.8 ± 41.4*	172.8 ± 58.2§
Fasting glucose (mg/dL)	89.6 ± 8.0	85.7 ± 20.6	151.8 ± 36.4*	135.8 ± 32.3
Fasting insulin (ng/mL)	0.72 ± 0.41	0.57 ± 0.72	1.63 ± 0.81*	2.10 ± 0.74
HOMA-IR	2.9 ± 1.8	2.0 ± 2.5	11.0 ± 6.7*	13.3 ± 6.6
Serum cholesterol (mg/dL)	90.5 ± 3.8	85.1 ± 4.9	174.8 ± 22.9*	263.0 ± 28.5§
Serum TG (mg/dL)	24.8 ± 6.8	26.0 ± 5.6	31.3 ± 5.9*	33.8 ± 8.2
Serum NEFA (mEq/L)	1.2 ± 0.0	1.1 ± 0.2	1.8 ± 0.1*	1.7 ± 0.3
Serum leptin (ng/mL)	3.9 ± 2.0	3.5 ± 2.7	30.2 ± 4.4*	31.6 ± 22.9

*BD* basal diet, *HFD* high-fat diet, *HOMA-IR* homeostasis model assessment of insulin resistance, *TG* triglyceride, *NEFA* nonesterified fatty acid

Data are expressed as the mean ± SEM

Significant differences exist between *eNOS+/+ and BD vs. *eNOS+/+* and HFD; §*eNOS+/+* and HFD vs. *eNOS-/-* and HFD for listed parameters at the *P* < .05 level

**Table 3:** Histological Scores of Livers using the NAFLD activity score (NAS)^11)^

			12-week model			
Item	Definition	Score	*eNOS+/+*	*eNOS-/-*	*eNOS+/+*	*eNOS-/-*
			BD	BD	HFD	HFD
Steatosis						
Grade	Parenchymal involvement					
	<5 %	0	8	8	0	0
	5%-33 %	1	0	0	6	2
	33%-66 %	2	0	0	2	6
	>66 %	3	0	0	0	0
		Average	0.00	0.00	1.25*	1.75§
Inflammation						
Lobular inflammation	Assessment of all inflammatory foci					
	No foci	0	8	8	5	1
	<2 foci per 200 × field	1	0	0	3	7
	2-4 foci per 200 × field	2	0	0	0	0
	>4 foci per 200 × field	3	0	0	0	0
Liver cell injury	Ballooning					
	None	0	8	8	0	0
	Few balloon cells	1	0	0	8	8
	Many cells/prominent ballooning	2	0	0	0	0
		Average	0.00	0.00	1.38*	1.88§
Fibrosis						
Stage	Method of Brunt					
	None	0	8	8	8	8
	Perivenular/perisinusoidal fibrosis	1	0	0	0	0
	Combine pericellular portal fibrosis	2	0	0	0	0
	Septal/bridging fibrosis	3	0	0	0	0
	Cirrhosis	4	0	0	0	0
		Average	0.00	0.00	0.00	0.00

*BD* basal diet, *HFD* high fat diet

Significant differences exist between *eNOS+/+ and BD vs. *eNOS+/+* and HFD; §*eNOS+/+* and HFD vs. *eNOS-/-* and HFD for listed parameters at the *P* < .05 level

### Deficiency of eNOS-derived NO increased lipid deposits in the liver and decreased the visceral fat volume in HFD-fed mice

In the NAFLD/NASH model, an increase in the body weight was observed after 12 weeks of HFD feeding, and no significant difference was observed between the *eNOS+/+* and HFD mice and the *eNOS-/-* and HFD mice (Table 2). The *eNOS-/-* and HFD mice had a significantly higher liver weight and a significantly lower visceral fat weight than then *eNOS+/+* and HFD mice; furthermore, the *eNOS-/-* and HFD mice had a significantly higher liver weight/body weight ratio and a significantly lower visceral fat weight/body weight ratio, compared with the *eNOS+/+* and HFD mice (Table 2).

As an indicator of the degree of fat infiltration in the liver, the liver-to-spleen attenuation ratio obtained using non-contrast CT images showed that hepatic steatosis was significantly severer in the *eNOS-/-* and HFD mice than in the *eNOS+/+* and HFD mice (Fig. 1a).

The visceral fat volume as quantified using CT imaging was significantly lower in the *eNOS-/-* and HFD mice than in the *eNOS+/+* and HFD mice (Fig. 1b).

An analysis of the parameters associated with metabolic syndrome showed no significant differences between the *eNOS+/+* and HFD mice and the *eNOS-/-* and HFD mice when the fasting plasma glucose level, fasting insulin level, HOMA-IR, serum TG level, serum NEFA level, and serum leptin level were compared. However, the serum cholesterol level was significantly higher in the *eNOS-/-* and HFD mice than in the *eNOS+/+* and HFD mice (Table 2).

### Deficiency of eNOS-derived NO exacerbated the pathogenesis of early-stage NASH in HFD-fed mice

Pathological examination of the liver showed that the *eNOS-/-* and HFD mice had severer hepatic steatosis and inflammation than the *eNOS+/+* and HFD mice (Fig. 2a and 2c). The liver TG content and the serum ALT levels were significantly higher in the *eNOS-/-* and HFD mice than in the *eNOS+/+* and HFD mice (Fig. 2b and Table 2). The liver tissue MPO and Sirius red staining examinations showed no significant positive findings (Fig. 2d and 2e), and the additional liver tissue naphthol AS-D chloroacetate esterase staining examination showed negative findings (data not shown). An analysis of the histological scores for the livers using NAS showed that the scores for the steatosis grade were significantly higher in the *eNOS-/-* and HFD mice than in the *eNOS+/+* and HFD mice; these results were consistent with the results of the CT scan examination. Furthermore, the scores for lobular inflammation and liver cell injury were significantly higher for the *eNOS-/-* and HFD mice than for the *eNOS+/+* and HFD mice (Table 3).

### Deficiency of eNOS-derived NO decreased the hepatic tissue blood flow

**Fig. 3:**
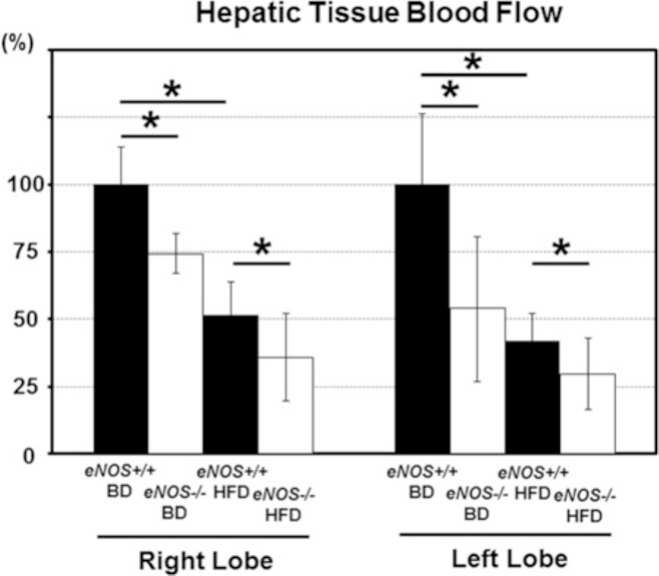
Analysis of hepatic talysis of hepatic tissue blood flow. The hepatic tissue blood flow measured using a noncontact-type laser Doppler blood flow meter was significantly lower in the *eNOS-/-* mice than in the *eNOS+/+* mice under each feeding condition (BD or HFD) in both hepatic lobes at 12 weeks. The hepatic tissue blood flow of the *eNOS+/+*/HFD mice was significantly lower than that of the *eNOS+/+*/BD mice. (Data were expressed as the mean ± SEM. *P < 0.05 represents a significant difference.)

Measurement of the hepatic tissue blood flow using a noncontact-type laser Doppler blood flow meter showed that a deficiency of eNOS-derived NO significantly decreased the hepatic tissue blood flow in both hepatic lobes under each dietary condition (Fig. 3). HFD feeding was also shown to significantly decrease the hepatic blood flow, since the value for the *eNOS+/+* and HFD mice was significantly lower than that of the *eNOS+/+* and BD mice (Fig. 3).

### Comparison of the mechanism associated with the pathogenesis of NAFLD between wild-type and eNOS-knockout mice under HFD conditions

**Fig. 4:**
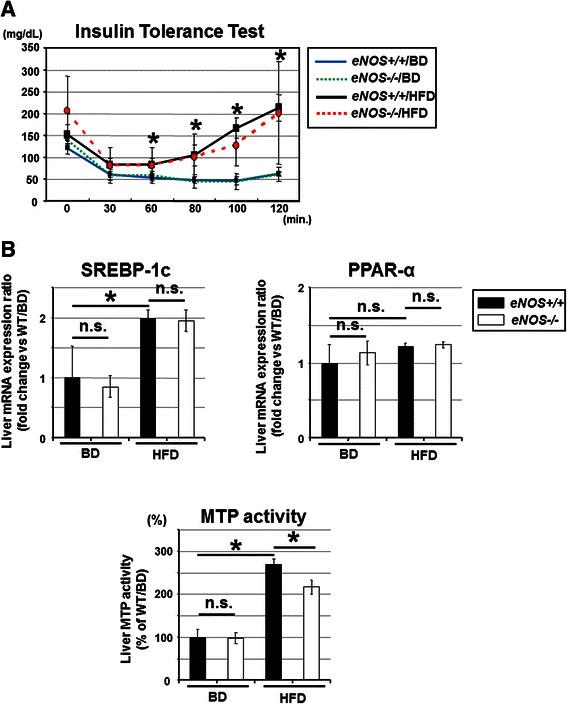
Analysis of the mechanism associated with the pathogenesis of NAFLD. **a** ITT results at 12 weeks showed no significant difference in the systemic response to insulin injection between the *eNOS+/+*/HFD mice and the *eNOS-/-*/HFD mice, while it was significantly lower in the *eNOS+/+*/HFD mice than in the *eNOS+/+*/BD mice, which were regarded as the control group. **b** The liver SREBP-1c and PPAR-α1 mRNA expression levels were comparable between the *eNOS-/-*/HFD mice and the *eNOS-/-*/HFD mice. The liver MTP activity level was significantly lower in the *eNOS-/-*/HFD mice than in the *eNOS+/+*/HFD mice at 12 weeks. The liver SREBP-1c mRNA expression level and the liver MTP activity level were significantly higher in the *eNOS+/+*/HFD mice than in the *eNOS+/+*/BD mice, which were regarded as the control group. (Data were expressed as the mean ± SEM. *P < 0.05, represents a significant difference; n.s. represents no significant difference.)

The ITT results at 12 weeks showed no significant difference in the systemic response to insulin injection between the *eNOS+/+* and HFD mice and the *eNOS-/-* and HFD mice, while it was significantly lower in the *eNOS+/+* and HFD mice than in the *eNOS+/+* and BD mice (Fig. 4a).

An analysis of the liver mRNA expression levels showed that no significant differences in the liver mRNA levels of SREBP-1c and PPAR-α1 were observed between the *eNOS+/+* and HFD mice and the *eNOS-/-* and HFD mice. Furthermore, the liver MTP activity level was significantly down-regulated in the *eNOS-/-* and HFD mice, compared with the *eNOS+/+* and HFD mice (Fig. 4b). As for the liver mRNA levels of other isoforms of NOS, no significant differences in the levels of either nNOS or iNOS were observed between the *eNOS+/+* and HFD mice and the *eNOS-/-* and HFD mice (data not shown).

## Discussion

NAFLD/NASH is presently the most common chronic liver disorder, displaying a wide spectrum of liver damage ranging from simple steatosis to steatohepatitis, advanced fibrosis, and cirrhosis. NAFLD/NASH is a complex disease with no simple causes. A new disease model has been proposed suggesting that multiple hits may act in parallel, resulting in liver inflammation; this model is known as the multiple parallel hits hypothesis [24]. A relationship between fatty liver changes and hepatic microcirculation has been described in several studies [25, 26]. Moreover, another study demonstrated that key signaling molecules mediating the metabolic actions of insulin were necessary for insulin to stimulate the production of NO and vasodilatation in human vascular endothelium [27]. Thus, we focused on the role of the *eNOS* gene in the pathogenesis of NAFLD/NASH; NO, produced by various isoforms of NOS, is an ubiquitous signaling molecule involved in the regulation of metabolic homeostasis, and eNOS, which the endothelium produces as a vasoactive substance, has been shown to serve important functions, including the regulation of regional blood flow, insulin resistance, and energy production [6, 28].

In the present study, we used an HFD-induced NAFLD/NASH mouse model with or without the *eNOS* gene. In our NAFLD/NASH model using HFD conditions, prominent hepatic steatosis and very mild liver inflammation were observed after 12 weeks, but typical fibrosis was not observed. In this “early-stage NAFLD/NASH” model, eNOS-derived NO changed the fat distributions in the liver and viscera of the mice. *eNOS*-knockout mice reportedly exhibit a clustering of symptoms belonging to the metabolic syndrome phenotype, such as body weight gain, hypertension, insulin resistance, and dyslipidemia [29]. These parameters of *eNOS*-knockout mice and wild-type mice reportedly differ according to diet, sex, and the study period [28–34]. In both the *eNOS*-knockout mice and the wild-type mice fed an HFD during the 12 weeks of our study period, obesity, insulin resistance, dyslipidemia and high serum leptin levels were observed, although these markers were comparable between the *eNOS*-knockout and the wild-type mice fed an HFD, with the exception of the serum cholesterol level. Hepatic steatosis and the increases in the serum ALT levels were significantly severer in the *eNOS*-knockout mice fed an HFD than in the wild-type mice fed an HFD, while the visceral fat volume was significantly lower in the *eNOS*-knockout mice fed an HFD, compared with the wild-type mice fed an HFD in this study.

The mechanism responsible for the changes in fat distribution in the liver and viscera of *eNOS*-knockout mice fed an HFD may be associated with the hepatic blood flow. Actually, *eNOS*-knockout mice fed an HFD exhibited a significantly lower hepatic tissue blood flow, compared with wild-type mice fed an HFD, with no significant difference in systemic insulin clearance observed between the *eNOS*-knockout mice and the wild-type mice fed an HFD. These results suggested that eNOS may play an important role in the progression of “early-stage NAFLD/NASH” not through insulin resistance, but through direct hepatic vascular action under the HFD condition. An analysis of factors associated with lipid metabolism in the liver showed that the liver MTP activity level was significantly lower in the *eNOS*-knockout mice fed an HFD than in the wild-type mice fed an HFD. MTP is a heterodimeric lipid transfer protein that is essential for very low density lipoprotein synthesis and transfer; a polymorphism of the MTP promoter reportedly leads to decreased MTP transcription and a greater intrahepatocellular triglyceride accumulation, thereby determining the susceptibility to NASH [35]. The down-regulation of hepatic MTP activity could lead to a decreased visceral fat volume by decreasing the outflow of lipids from the liver. However, we could not determine the relation between eNOS and the liver MTP activity in our study model, and few studies have examined this point.

eNOS is known to serve important functions, including the regulation of vascular tone and regional blood flow [6]. On the other hand, hepatic tissue blood flow and hepatic microcirculation have been shown to be strongly associated with the progression of NAFLD/NASH [7, 36–38]. In NASH liver, ballooning, which consists of fat-laden swollen hepatocytes, causes sinusoidal distortion, reducing the intrasinusoidal volume and microvascular blood flow [36–38]. In our *eNOS*-knockout mouse model, while wild-type mice fed an HFD had a significantly lower hepatic tissue blood flow than the wild-type mice fed a BD, the *eNOS*-knockout mice fed a BD had a significantly lower hepatic blood flow than the wild-type mice fed a BD. These results suggested that the decrease in hepatic blood flow in the *eNOS*-knockout mice fed an HFD was not only a secondary effect of lipid accumulation and compression of the sinusoid, but was also caused by the *eNOS* gene deficiency.

Sheldon et al. reported that chronic NOS inhibition via N^ω^-nitro-L-arginine methyl ester in obese Otsuka Long-Evans Tokushima Fatty rats reduced hepatic mitochondrial respiration, leading to increased hepatic triacylglycerol accumulation, and increased hepatic inflammation, although the specific mechanism remained unclear [8]. They did not examine blood flow in the hepatic tissue; however, the mechanism related to exacerbated early-stage NAFLD pathogenesis under the condition of eNOS deficiency might be associated with the function of hepatic mitochondrial respiration.

Regarding insulin resistance, no significant differences in systemic insulin resistance, such as the HOMA-IR or ITT results, were observed between the wild-type and *eNOS*-knockout mice under the HFD conditions in this model. However, direct hepatic insulin responsiveness was not assessed in this study.

Although the present study revealed a change in the fat distribution induced by both the *eNOS* gene and the HFD in mice in an “early-stage NAFLD/NASH” model, longer-term analysis may detect a different phenomenon in “advanced-stage NAFLD/NASH.” A potential limitation of the current study includes the use of systemic *eNOS*-knockout mice. In particular, studies using liver-specific *eNOS*-knockout mice fed an HFD or *eNOS* transgenic mice fed an HFD in a long-term HFD feeding analysis are needed. Further studies are needed to verify hepatic mitochondrial respiration markers and hepatic inflammation states, and to examine the relationship between the changes in hepatic blood flow and the degree of liver injury in order to determine the exact underlying mechanism.

## Conclusions

In conclusion, a deficiency of eNOS-derived NO may change the fat distributions in the liver and viscera, thereby promoting the progression of disease in an HFD-induced, early-stage NASH mouse model by changing the hepatic tissue blood flow. This report is also the first study to examine the fat distribution and the pathogenesis of NAFLD/NASH using an imaging procedure in an NAFLD/NASH mouse model with or without the *eNOS* gene.

## Abbreviations

ALT: alanine aminotransferase
BD: basal diet
Chol: cholesterol
CT: computed tomography
eNOS: endothelial nitric oxide synthase
HFD: high-fat diet
HOMA-IR: homeostasis model assessment of IR
iNOS: inducible nitric oxide synthase
IR: insulin resistance
ITT: insulin tolerance test
MPO: myeloperoxidase
MTP: microsomal triglyceride transfer protein
NAFL: non-alcoholic fatty liver
NAFLD: non-alcoholic fatty liver disease
NAS: NAFLD activity score
NASH: non-alcoholic steatohepatitis
NEFA: nonesterified fatty acid
nNOS: neuronal nitric oxide synthase
NO: nitric oxide
NOS: nitric oxide synthase
PPAR-α1: peroxisome proliferators-activated receptor alpha1
SREBP-1c: sterol regulatory element binding protein-1c
TG: triglyceride
